# Boosting Methane Combustion Performance and Stability of Spherical Mesoporous Silica (KCC-1)-Supported Pd Catalysts by Modifying with CeO_2_

**DOI:** 10.3390/nano16040231

**Published:** 2026-02-11

**Authors:** Kaien Feng, Jinxiong Tao, Zhiquan Hou, Yuxi Liu, Jiguang Deng, Lu Wei, Zhen Wei, Lin Jing, Hongxing Dai

**Affiliations:** 1 Beijing Key Laboratory for Green Catalysis and Separation, State Key Laboratory of Materials Low-Carbon Recycling, Laboratory of Catalysis Chemistry and Nanoscience, Department of Chemical Engineering and Technology, College of Materials Science and Engineering, Beijing University of Technology, Beijing 100124, China; 2 Key Laboratory of Beijing on Regional Air Pollution Control, College of Environmental Science and Engineering, Beijing University of Technology, Beijing 100124, China

**Keywords:** spherical mesoporous silica, ceria modification, supported Pd catalyst, methane combustion, high-temperature stability, water resistance

## Abstract

In the present work, 1.92 wt% Pd/9.68 wt% CeO_2_/spherical mesoporous silica (denoted as 1.92Pd/9.68CeO_2_/KCC-1) and 1.96 wt% Pd/KCC-1 (denoted as 1.96Pd/KCC-1) catalysts were prepared. It was found that the 1.92Pd/9.68CeO_2_/KCC-1 sample exhibited an excellent catalytic activity for methane combustion, which was much better than that of the 1.96Pd/KCC-1 sample. In addition, the 1.92Pd/9.68CeO_2_/KCC-1 sample possessed good high-temperature stability and water resistance. The enhanced methane combustion performance of 1.92Pd/9.68CeO_2_/KCC-1 was mainly attributed to the good dispersion of Pd species and the stabilization of the active Pd^2+^ species and generation of more reactive oxygen species by CeO_2_ modification. This work offers new insights into developing methane combustion catalysts with low-temperature catalytic performance and high-temperature stability.

## 1. Introduction

Methane, as the primary component of natural gas, has been widely utilized in industries and transportation activities [1]. Natural gas-driven vehicles have been proven to be an alternative to gasoline- and diesel-powered vehicles, in which natural gas offers higher fuel efficiency and less pollutant emissions [2,3]. However, methane is the world’s second-largest greenhouse gas with high global warming potential and a short atmospheric lifetime [4,5]. Therefore, effectively reducing unburned methane emissions from vehicle exhaust has become a big challenge [6,7]. Strictly controlling methane emissions offers three environmental benefits of (i) mitigating global warming; (ii) promoting the economic value of energy resource utilization, and (iii) achieving synergistic pollution control.

Among numerous methane treatment methods, catalytic oxidation is considered one of the most environmentally friendly approaches due to its low energy consumption and high removal efficiency [8,9]. Up to now, a large number of studies have been conducted on methane combustion over the catalysts, including precious metal (e.g., Pd [10,11,12], Pt [13,14], and Au [15]) catalysts and non-noble metal (including Al [16,17], Co [18], and Ni [19,20]) catalysts. Among these catalysts, the supported Pd-based catalysts have been demonstrated to be the most efficient for methane combustion. However, the Pd nanoparticles (NPs) in supported Pd-based catalysts can be easily sintered at high temperatures, thus leading to deactivation of the catalysts.

Suitable support can prevent the thermal decomposition of PdO*_x_* and the sintering of Pd NPs at high temperatures, thereby enhancing the stability of the active PdO*_x_* phase. Examples include metal oxides [21,22,23,24], zeolites [25,26,27], mesoporous silica [28,29], and metal–organic frameworks (MOFs) [30,31], in which there are pore-confined structures that can effectively prevent the sintering and agglomeration of noble metals. Spherical mesoporous silica (KCC-1) is a silica material featuring an open mesoporous structure. KCC-1 possesses excellent properties (e.g., high surface area, regular pores, and good thermal stability), hence being widely employed as a support for various catalytic reactions [32,33]. This unique feature enhances the dispersion of noble metal NPs, the stability of supported noble metal catalysts, and the facilitation of reactants diffusion. Singh and Polshettiwar [34] found that compared to MCM-41, KCC-1 exhibited a higher surface area and a larger pore volume due to its distinctive tubular pore structure, thereby increasing the mass transfer efficiency. Hamid et al. [35] reported that KCC-1 possessed significantly higher basicity than MCM-41, demonstrating a fivefold higher catalytic activity than MCM-41 in CO_2_ methanation. It is evident that the spherical mesoporous structure of KCC-1 significantly increases the surface area, ensuring complete penetration of the loaded noble metal NPs into its pores, resulting in uniform and well-dispersed NPs that are beneficial for the enhancement in catalyst activity.

CeO_2_ is an abundant, cost-effective, and structurally stable rare-earth oxide, which has been widely used as a support for loading noble metal NPs due to its good thermal stability, large oxygen storage capacity, and unique redox property [36,37]. For instance, He et al. [38] introduced CeO_2_ nanoislands onto dendritic mesoporous SiO_2_ and selected Pt as the active component, and the as-obtained catalyst achieved a 1.5-fold increase in propane combustion efficiency at 210 °C compared to the CeO_2_-free counterpart. Tan et al. [39] investigated a core–shell catalyst featuring Pd/CeO_2_ loaded on a SiO_2_ shell for methane combustion, and observed that after seven recycles, 90% methane conversion was achieved over the core–shell catalyst at 385 °C. In comparison, the traditionally prepared Pd/CeO_2_ catalyst required a higher temperature (440 °C) to reach the same methane conversion. Feng et al. [40] prepared a highly dispersed platinum (Pt/CeO*_x_*/DMS) catalyst for CO oxidation, and claimed that the *T*_90%_ for CO oxidation over Pt/CeO*_x_*/DMS was decreased by 16 °C compared to that over Pt/DMS, and the former maintained excellent catalytic performance even after 8 h of on-stream reaction at 500 °C. Therefore, doping CeO_2_ to KCC-1 can leverage its oxygen storage capacity to provide the additional active adsorbed oxygen species and enhance the interaction between noble metal NPs and CeO_2_, thereby improving both catalytic activity and stability of the supported noble metal catalysts.

In this work, we prepared a mesoporous KCC-1-supported palladium-based catalyst modified with ceria (1.92Pd/9.68CeO_2_/KCC-1) and made a comparison on catalytic methane combustion activity of 1.92Pd/9.68CeO_2_/KCC-1, 1.92Pd/KCC-1, and 10.23CeO_2_/KCC-1. Compared with traditional catalysts, the unique structure of KCC-1 enables CeO_2_ to attach more readily to the surface of the support during the modification process. This allows for the provision of more oxygen species to facilitate the methane oxidation, resulting in excellent catalytic activity at lower temperatures of the CeO_2_-doped sample. The physicochemical properties of these materials were characterized, and their catalytic activities, high-temperature stability, and water resistance for methane combustion were investigated in detail.

## 2. Experimental

### 2.1. Synthesis of KCC-1

The synthesis method described in the literature [41] was modified to some extent. First, KCC-1 with a specific morphology was synthesized using the hydrothermal method. A total of 1.6 g of hexadecyltrimethylammonium bromide and 0.96 g of urea were dissolved in 48 mL of deionized water to form Solution A. Concurrently, 2.4 mL of 1-pentanol and 4.3 mL of tetraethoxysilane were mixed in 48 mL of cyclohexane under stirring for 30 min to form Solution B. Then, Solution B was added dropwise to Solution A under stirring for 30 min. The resulting mixed solution was placed in a PTFE-sealed autoclave and heated in an oven at 120 °C for 4 h. After the products were centrifuged at 10,000 rpm, the resulting material was dried in an oven at 80 °C for 12 h, calcined in air and a muffle furnace at a heating rate of 5 °C/min from room temperature to 550 °C and maintained for 6 h, thus obtaining the KCC-1 support.

### 2.2. Preparation of Pd/CeO_2_/KCC-1

A total of 0.5 g of the KCC-1 support was added to 8 mL of deionized water. Then, 0.433 g of Ce(NO_3_)_3_·6H_2_O was added under stirring for 6 h. The mixture was transferred to a muffle furnace after rotary evaporation and calcined in air atmosphere at a ramp of 5 °C/min from room temperature to 400 °C and kept for 2 h, thus preparing the CeO_2_/KCC-1 catalyst. Pd was loaded using a polyvinyl alcohol (PVA)-protected NaBH_4_ reduction method. First, the PVA solution was stirred until a dense foam formed; then, 8.33, 12.49 or 16.66 mL (corresponding to theoretical Pd loadings of 0.5, 1.5, and 2.0 wt%) of the PdCl_2_ aqueous solution (2 g/L) was added under stirring for 1 h; finally, the NaBH_4_ solution was rapidly added under an ice-bath condition to ensure formation of the uniform solution color. Subsequently, 0.5 g of CeO_2_/KCC-1 was added to the above Pd-containing solution and stirred for 6 h. The mixture was filtered in vacuum and dried in an oven at 80 °C overnight. The dried sample was then transferred to a muffle furnace for calcination in an air atmosphere at a heating rate of 5 °C/min from room temperature to 500 °C and maintained for 2 h, hence obtaining the *x*Pd/CeO_2_/KCC-1 (theoretical Pd loadings (*x*) = 0.5, 1.5, and 2.0 wt%) samples.

The method for the preparation of *x*Pd/KCC-1 (theoretical Pd loadings (*x*) = 2.0 wt%) was identical to that for the preparation of *x*Pd/CeO_2_/KCC-1 (theoretical Pd loadings (*x*) = 0.5, 1.5, and 2.0 wt%), except that KCC-1 was used as the support and Ce(NO_3_)_3_·6H_2_O was not added.

### 2.3. Catalyst Characterization

Physicochemical properties of all the samples were determined using the following techniques: inductively coupled plasma–atomic emission spectroscopy (ICP–AES), X-ray diffraction (XRD), scanning electron microscopy (SEM), transmission electron microscopy (TEM), high-angle annular dark field–scanning transmission electron microscopy (HAADF–STEM) and EDX element mappings, laser Raman spectroscopy (Raman), nitrogen adsorption–desorption (BET), X-ray photoelectron spectroscopy (XPS), hydrogen temperature-programmed reduction (H_2_-TPR), methane temperature-programmed reduction (CH_4_-TPR), methane temperature-programmed surface reaction (CH_4_-TPSR), and in situ diffuse reflectance infrared Fourier transform spectroscopy (in situ DRIFTS). The detailed measurement procedures are described in the Supplementary Materials.

### 2.4. Catalytic Activity Assessment

Catalytic activities of the samples for methane combustion were assessed at atmospheric pressure using continuous-flow fixed-bed quartz microreactor (6.0 mm in inner diameter). A thermocouple was positioned at the center of the catalyst bed to monitor the reaction temperature. Reactants and products were analyzed online using gas chromatography (GC-2010, Shimadzu, Japan) with a stable wax-DA column (30 m in length), a Carboxen 1000 column (3 m in length), and a flame ionization detector (FID). For testing, 0.05 g of the catalyst (40–60 mesh) was diluted with 0.25 g of quartz sand (40–60 mesh). The reactant mixture composition was (10,000 ppm CH_4_ + 20.0 vol% O_2_ + N_2_ (balance)). A total reactant mixture flow rate of 16.7 mL/min was passed through the reactor, arising a space velocity (SV) of approximately 20,000 mL/(g h). Before conducting the activity test, the catalyst was pretreated at 300 °C for 1 h with an oxygen flow of 20 mL/min. For the water-resistance test, a water vapor concentration of 5.0 vol% was generated using a water saturator at 37 °C. Under identical conditions, long-term stability testing was conducted at 370 °C and SV = 20,000 mL/(g h). Catalytic activity was evaluated by adopting the temperatures (*T*_10%_, *T*_50%_, and *T*_90%_) corresponding to methane conversions of 10, 50, and 90%. Methane conversion (*X*_CH4_) was calculated according to Equation (1):

*X*_CH4_ = ([*c*_CH4_]_in_ − [*c*_CH4_]_out_)/[*c*_CH4_]_in_ × 100% (1)

where [*c*_CH4_]_in_ and [*c*_CH4_]_out_ represent the inlet and outlet methane concentrations, respectively.

Specific methane reaction rate (*r*_CH4_) and turnover frequency (TOF_Pd_) were calculated according to Equations (2) and (3), respectively:

(2) $$r_{CH4}=\frac{[c_{CH4}]\times X_{CH4}\times F\times 10^{3}}{R\times T\times m_{cat}\times 60}(\mu mol/(g_{cat}\;s))$$

(3) $${TOF}_{Pd}=\frac{[c_{CH4}]\times X_{CH4}\times F\times M_{Pd}\times 10^{3}}{R\times T\times m_{cat}\times L_{Pd}\times D_{Pd}\times 60}(s^{-1})$$

where [*c*_CH4_], *F*, *T*, *m*_cat_, *L*_Pd_, *R*, *M*_Pd_, and *D*_Pd_ are the methane concentration, total flow rate, temperature (298 K), catalyst mass, Pd loading, gas constant, Pd molecular weight, and Pd dispersion, respectively.

## 3. Results and Discussion

### 3.1. Crystal Phases and Textural Properties

**Figure 1:**
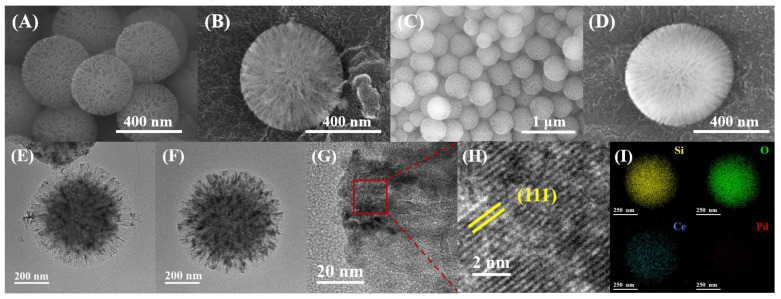
SEM images of (**A**) KCC-1, (**B**) 1.96Pd/KCC-1 and (**C**,**D**) 1.92Pd/9.68CeO_2_/KCC-1, and (**E**) TEM image of 1.96Pd/KCC-1, and (**F**–**H**) TEM images and (**I**) EDX elemental mappings of 1.92Pd/9.68CeO_2_/KCC-1.

The actual loadings of Pd and CeO_2_ in the samples were determined using the ICP–AES analysis technique, as listed in Table 1. The actual contents of Pd and Ce in the Pd/CeO_2_/KCC-1 samples were 0.97 and 9.68 wt%, 1.44 and 9.68 wt%, and 1.92 and 9.68 wt%, respectively, while the Pd content in the Pd/KCC-1 sample was 1.96% and the actual Ce content in the CeO_2_/KCC-1 sample was 10.23 wt%. The overall morphology of KCC-1 was observed using the SEM technique. The as-prepared KCC-1 support exhibited a uniformly spherical porous structure with a diameter of approximately 400 nm (Figure 1A), confirming the successful synthesis of a high-surface-area support. Figure 1B–D shows SEM images of the 1.96Pd/KCC-1 and 1.92Pd/9.68CeO_2_/KCC-1 samples. The morphology remained unchanged after loading of Pd and CeO_2_. Additionally, KCC-1 (Figure 1A and Figure S1A) and 1.96Pd/KCC-1 (Figure 1B and Figure S1B) samples displayed the same morphology, with no significant variations in spherical particle size. The HAADF–STEM technique was further employed to investigate element distributions of the samples at the atomic scale. Figure 1E–H shows TEM images of the as-prepared 1.96Pd/KCC-1 and 1.92Pd/9.68CeO_2_/KCC-1 samples, which reveal a spherical framework extending from the center to the periphery. The particle sizes of the two samples were in the range of 300–400 nm. Additional TEM images of the 1.92Pd/9.68CeO_2_/KCC-1 sample are also shown in Figure S2. Upon magnification, CeO_2_ NPs were clearly visible, with the (111) interplanar spacing being measured to be 0.322 nm. It can be seen that there are Pd NPs uniformly dispersed on the sample surface, and the average Pd particle sizes in the 1.92Pd/9.68CeO_2_/KCC-1 and 1.96Pd/KCC-1 samples were 3.0 ± 0.7 and 5.0 ± 0.8 nm (Figure S3), respectively, which were obtained by statistically analyzing 100 Pd NPs on the TEM images of the two samples. In addition, the EDX elemental mappings (Figure 1I) demonstrate that both Ce and Pd are highly dispersed on the support surface.

**Figure 2:**
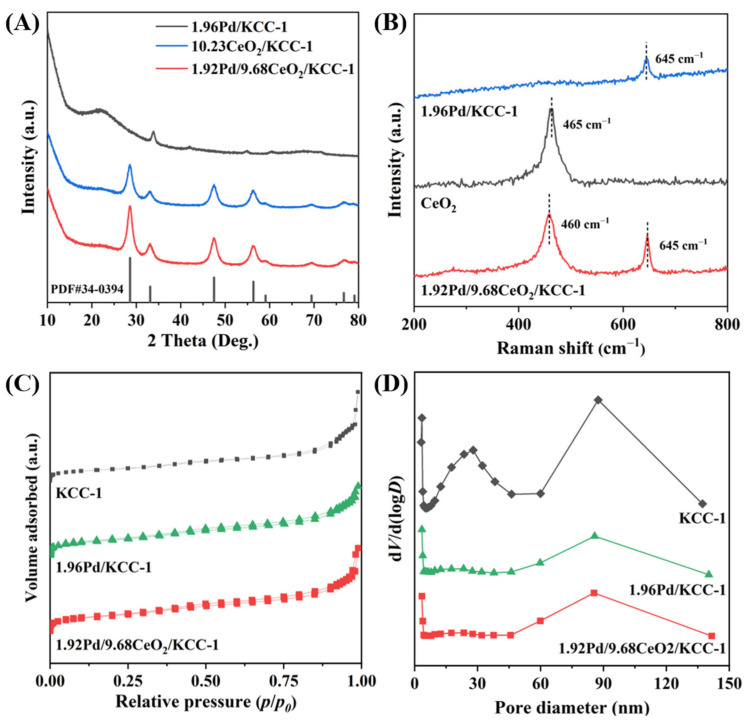
(**A**) XRD patterns of 1.96Pd/KCC-1, 10.23CeO_2_/KCC-1, and 1.92Pd/9.68CeO_2_/KCC-1, (**B**) Raman spectra of CeO_2_, 1.96Pd/KCC-1, and 1.92Pd/9.68CeO_2_/KCC-1, and (**C**) nitrogen adsorption–desorption isotherms and (**D**) pore-size distributions of KCC-1, 1.96Pd/KCC-1, and 1.92Pd/9.68CeO_2_/KCC-1.

Crystal phase compositions of the samples were determined using the XRD technique. Figure 2A shows XRD patterns of the 1.92Pd/9.68CeO_2_/KCC-1, 1.96Pd/KCC-1, and 10.23CeO_2_/KCC-1 samples. The diffraction peaks of CeO_2_ appeared at 2*θ* = 28.5°, 33.5°, 47.5°, 56.3°, 59.1°, 69.4°, 76.7°, and 79.1°. A comparison in XRD patterns of the as-fabricated samples with that (JCPDS PDF# 34-0394 [42]) of the standard CeO_2_ sample reveals that the as-synthesized CeO_2_ sample is of a fluorite-type cubic structure [43]. It should be noted that there is a weak diffraction signal at 2*θ* = ca. 33.5° for the 1.96Pd/KCC-1 and 1.92Pd/9.68CeO_2_/KCC-1 samples, which could be assigned to the (110) crystal face of tetragonal PdO (JCPDS PDF# 41-1107). It is well-known that XRD is an effective tool to analyze the crystal structure of a bulk material. The Pd species in the 1.96Pd/KCC-1 and 1.92Pd/9.68CeO_2_/KCC-1 samples were present in the form of bulk PdO, but their surface Pd^0^ and Pd^2+^ compositions were different, as confirmed by the subsequent Pd 3d XPS results. According to the Scherrer formula, the Pd crystallite sizes in 1.92Pd/9.68CeO_2_/KCC-1 and 1.96Pd/KCC-1 were calculated to be 2.89 and 4.63 nm, respectively.

Figure 2B displays Raman spectra of the 1.92Pd/9.68CeO_2_/KCC-1, 1.96Pd/KCC-1, and CeO_2_ samples. No vibration bands of the KCC-1 support were detected within the scanned range. The characteristic band at 465 cm^−1^ originated from the F_2g_ mode of oxygen atoms symmetrically vibrating around Ce in CeO_2_, which was attributable to the fluorite-type cubic CeO_2_ structure [44]. The characteristic band at 645 cm^−1^ was ascribed to the B_1g_ vibration of the PdO phase induced by Pd loading. Therefore, the Raman spectra of the samples reveal that the characteristic bands at 645 and 465 cm^−1^ are due to the PdO and CeO_2_ species, respectively, which were confirmed by the subsequent XPS characterization results. After Pd loading, the band at 645 cm^−1^ was shifted toward a lower wavenumber, which is likely attributed to the pronounced interaction between Pd and CeO_2_ [45,46].

N_2_ adsorption–desorption isotherms and pore-size distributions of the samples were measured using the BET technique, as shown in Figure 2C,D and Table 1. All the samples exhibited an IV-typed isotherm, with a H3-typed hysteresis loop appearing at a relative pressure (*p*/*p*_0_) of 0.8–1.0, suggesting a stacked mesoporous architecture. Table 1 summarizes the surface areas and pore volumes of the samples. The surface areas of the KCC-1, 1.96Pd/KCC-1, 10.23CeO_2_/KCC-1, and 1.92Pd/9.68CeO_2_/KCC-1 samples were 555, 480, 367, and 304 m^2^/g, respectively, and the drop in surface area of the CeO_2_-loaded samples might be due to the partial blocking of mesoporous pores by CeO_2_ [29]. It should be pointed out that the *x*Pd/9.68CeO_2_/KCC-1 samples with different Pd loadings (0.97Pd/9.68CeO_2_/KCC-1, 1.44Pd/9.68CeO_2_/KCC-1, and 1.92Pd/9.68CeO_2_/KCC-1) exhibited a close surface area (301–304 m^2^/g). Pore sizes of all the samples determined by the BJH method were in the range of 9–13 nm, confirming formation of a mesoporous structure [19]. It is noted that the macropores of about 30 and/or 90 nm stem from the aggregation of spherical KCC-1 particles in all the samples.

### 3.2. Reducibility, Surface Properties, and Adsorption–Desorption Behavior

**Figure 3:**
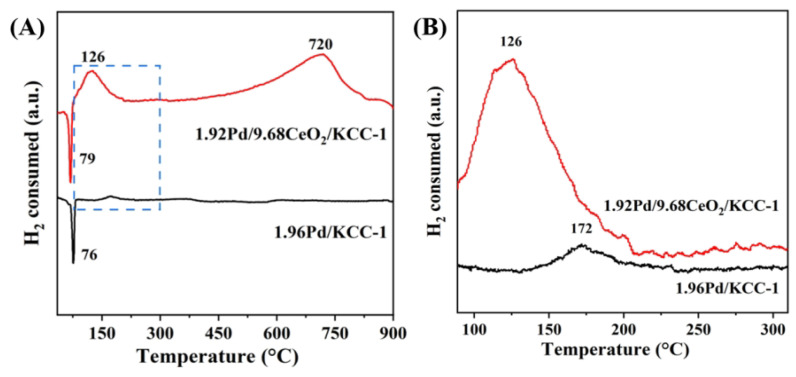
(**A**) H_2_-TPR profiles and (**B**) partially enlarged H_2_-TPR profiles (in the range of 100–300 °C) of the 1.92Pd/9.68CeO_2_/KCC-1 and 1.96Pd/KCC-1 samples.

Reducibility of the samples was investigated using the H_2_-TPR technique, with their profiles being shown in Figure 3A. The reverse low-temperature reduction peak at 76 or 79 °C of the 1.96Pd/KCC-1 or 1.92Pd/9.68CeO_2_/KCC-1 sample stemmed from the desorption of hydrogen adsorbed at the Pd sites. For the 1.92Pd/9.68CeO_2_/KCC-1 sample, a reduction peak was observed at 126 °C, which was the removal of adsorbed oxygen species and the reduction of PdO. Comparing the peak at 172 °C due to the reduction of PdO for the 1.96Pd/KCC-1 sample (Figure 3B), the intensity of the peak at 126 °C for the 1.92Pd/9.68CeO_2_/KCC-1 sample was much stronger and its appearance temperature was lower by 46 °C, demonstrating that the doping of CeO_2_ favored the improvement in the low-temperature reducibility of the sample. There were the adsorbed oxygen species at the oxygen vacancies of CeO_2_ in 1.92Pd/9.68CeO_2_/KCC-1 and the atomic hydrogen species adsorbed on the Pd nanoparticles in the CeO_2_-doped KCC-1-supported Pd sample during the H_2_-TPR experiment favored the reduction of the adsorbed oxygen and PdO species, hence improving the low-temperature reducibility of the sample. The bigger reduction peak at 126 °C for the 1.92Pd/9.68CeO_2_/KCC-1 sample than that at 172 °C for the 1.96Pd/KCC-1 sample due to the reduction of PdO suggests that there is a much higher amount of Pd^2+^ species in the former than in the latter since the H_2_-TPR technique was used to determine the reduction behaviors of the bulk samples, although the surface Pd^2+^/(Pd^0^ + Pd^2+^) molar ratio on 1.92Pd/9.68CeO_2_/KCC-1 was slightly higher than that on 1.96Pd/KCC-1 (Table 1). Additionally, there was also a reduction peak at 720 °C for the 1.92Pd/9.68CeO_2_/KCC-1 sample, which was assigned to reduction of the Ce^4+^ to Ce^3+^ species [47]. Meanwhile, the peak at the high temperature was not observed for the 1.96Pd/KCC-1 sample without the doping of CeO_2_. The hydrogen consumption of the samples was determined through quantification of the reduction peaks in their H_2_-TPR profiles. The hydrogen consumption (0.171 mmol/g_cat_) of the 1.92Pd/9.68CeO_2_/KCC-1 sample was much higher than that (0.032 mmol/g_cat_) of 1.96Pd/KCC-1 sample (Table 1).

**Figure 4:**
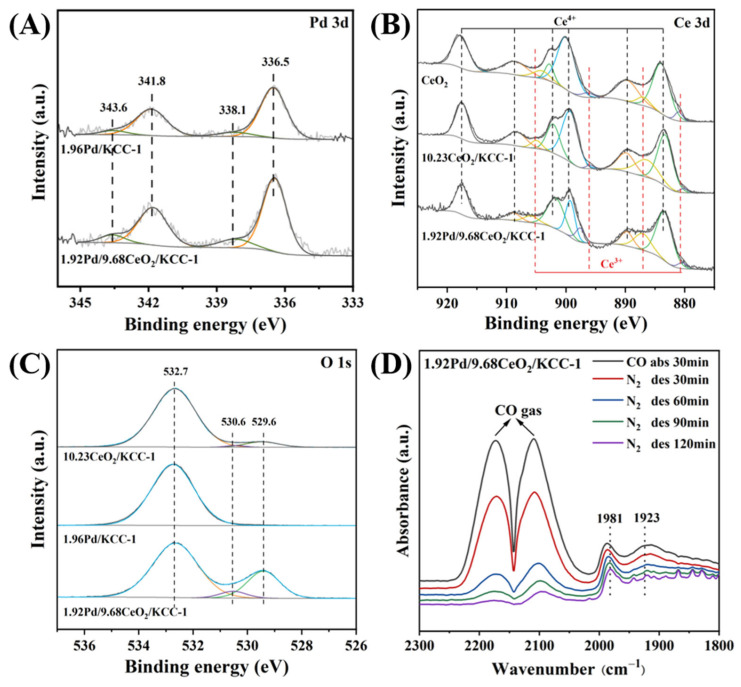
(**A**) Pd 3d, (**B**) Ce 3d, and (**C**) O 1s XPS of the 1.92Pd/9.68CeO_2_/KCC-1, 1.96Pd/KCC-1, 10.23CeO_2_/KCC-1, and CeO_2_ samples, and (**D**) in situ CO-DRIFTS spectra of the 1.92Pd/9.68CeO_2_/KCC-1 sample.

To investigate the relationship between surface properties (elemental compositions, metal valence states, and oxygen species) and catalytic performance of the samples, their O 1s, Ce 3d, and Pd 3d XPS spectra were recorded, as shown in Figure 4. The 1.92Pd/9.68CeO_2_/KCC-1 or 1.96Pd/KCC-1 sample exhibited an asymmetric peak in the Pd 3d XPS spectrum, which was decomposed into two components at binding energies (BEs) of 336.5 and 338.1 eV, corresponding to the surface Pd^0^ and Pd^2+^ species [48]. The Pd^2+^/(Pd^0^ + Pd^2+^) molar ratio (14.22) on 1.92Pd/9.68CeO_2_/KCC-1 was much higher than that (10.50) on 1.96Pd/KCC-1 (Table 1). Figure S4 shows Pd 3d XPS spectra of the *x* wt% Pd/9.68CeO_2_/KCC-1 samples at different Pd loadings. The Pd^2+^/(Pd^0^ + Pd^2+^) molar ratio on the surface of the supported Pd samples increased gradually from 12.49 to 14.22 with the rise in Pd loading from 0.97 to 1.92 wt%. An analysis on the Ce 3d XPS spectra reveals that there are the Ce 3d_3/2_ and Ce 3d_5/2_ final states. Six components at BEs of 883.3, 889.2, 899.6, 902.4, 908.7, and 917.9 eV were attributed to the surface Ce^4+^ species, whereas the two components at BEs of 880.7, 887.2, 896.1 and 905.2 eV were assigned to the surface Ce^3+^ species [49]. Since Ce exists in two distinct oxidation states, the formation of Ce^3+^ implies the generation of oxygen vacancies. That is to say, the CeO_2_-doped samples possess excellent oxygen storage and release capabilities. The Ce^3+^/(Ce^3+^ + Ce^4+^) molar ratio can be used to evaluate the concentration of Ce^3+^ on the sample surface by quantitatively analyzing the Ce^3+^ component peaks in Ce 3d XPS spectra of the samples. It is found that the surface Ce^3+^/(Ce^3+^ + Ce^4+^) molar ratio on 1.92Pd/9.68CeO_2_/KCC-1 is 24.16, higher than those (22.90 and 23.15) of the samples with other Pd loadings (see Table 1). A higher amount of Ce^3+^ species means a higher amount of oxygen vacancies, in which a more amount adsorbed oxygen species could be generated and the formed adsorbed oxygen species could facilitate stabilization of the active PdO phase, thereby enhancing the catalytic activity for methane combustion.

For O 1s XPS spectra of the 1.92Pd/9.68CeO_2_/KCC-1, 1.96Pd/KCC-1, and 10.23CeO_2_/KCC-1 samples, there were three components at BEs of 529.6, 530.6, and 532.7 eV. The peak at a BE of 532.7 eV was due to the bridging oxygen (Si–O–Si) species in the SiO_2_ structure of the KCC-1 support. The other two peaks at BEs of 529.6 and 530.6 eV corresponded to the surface lattice oxygen (O_latt_) and adsorbed oxygen (O_ads_) on the sample, respectively [50]. As can be seen from Table 1, the O_ads_/O_latt_ molar ratio (20.13) on 1.92Pd/9.68CeO_2_/KCC-1 was higher than that (17.66) on 10.23CeO_2_/KCC-1; furthermore, the O_ads_/O_latt_ molar ratios (18.01–20.13) on the *x*Pd/9.68CeO_2_/KCC-1 (*x* = 0.97–1.92 wt%) samples were close. It should be noted that almost no O_ads_ species are detected on the surface of the 1.96Pd/KCC-1 sample.

The precious metal loading state of the catalyst was investigated using in situ CO-DRIFTS characterization technique, as shown in Figure 4D. Two absorption bands at 1981 and 1923 cm^−1^ were observed for the 1.92Pd/9.68CeO_2_/KCC-1 sample, which were attributed to the CO adsorbed in a bridged configuration at the Pd site. Similarly, two absorption bands at 1983 and 1918 cm^−1^ were observed for the 1.96Pd/KCC-1 sample (Figure S5). The loaded Pd on 1.96Pd/KCC-1 might be mainly present in the form of clusters. After loading CeO_2_, the number of clusters increased, which was beneficial for the enhancement in methane combustion activity.

### 3.3. Catalytic Performance

**Figure 5:**
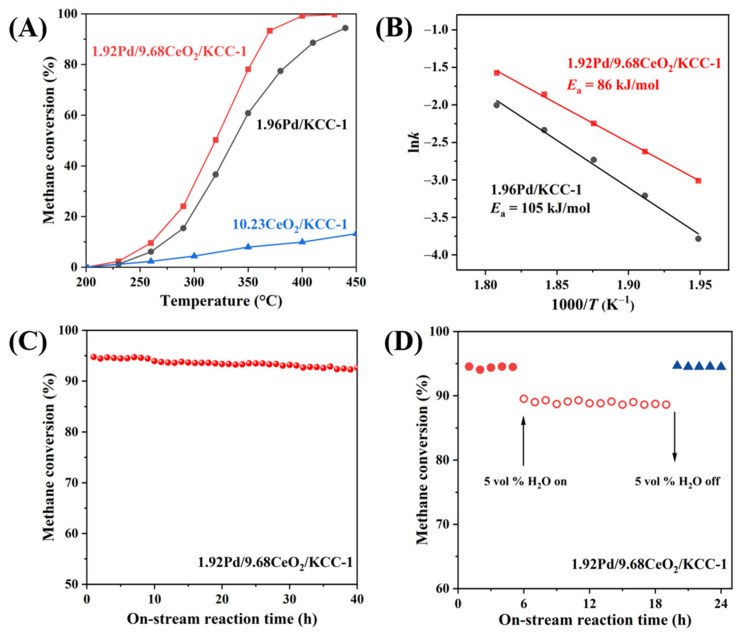
(**A**) Catalytic activity for CH_4_ combustion versus temperature over 10.23CeO_2_/KCC-1, 1.96Pd/KCC-1, and 1.92Pd/9.68CeO_2_/KCC-1, (**B**) ln*k* versus inverse temperature of 1.96Pd/KCC-1 and 1.92Pd/9.68CeO_2_/KCC-1 for methane combustion, (**C**) long-term stability of 1.92Pd/9.68CeO_2_/KCC-1 for methane combustion, and (**D**) effect of 5 vol% water vapor on methane combustion over 1.92Pd/9.68CeO_2_/KCC-1 at 370 °C and SV = 20,000 mL/(g h).

To compare catalytic activities of the samples, we use the reaction temperatures (*T*_50%_ and *T*_90%_) required for achieving methane conversions of 50 and 90%, respectively. Figure S6 shows catalytic activities of the *x*Pd/9.68CeO_2_/KCC-1 (*x* = 0.97, 1.44, and 1.92 wt%) samples for methane combustion under the conditions of (10,000 ppm CH_4_ + 20.0 vol% O_2_ + N_2_ (balance)) and SV = 20,000 mL/(g h). Methane conversion increased with increasing the Pd loading. Figure S7 shows catalytic activities of the 1.92Pd/yCeO_2_/KCC-1 (Ce loading (*y*) = 5.27, 9.68, and 18.81 wt%) samples for methane combustion. Apparently, the 1.92Pd/9.68CeO_2_/KCC-1 sample performed the best for methane combustion among the three samples with different Ce loadings. Therefore, the 1.92Pd/9.68CeO_2_/KCC-1 sample was selected for further investigation. The 1.92Pd/9.68CeO_2_/KCC-1 sample with a Pd loading (*x*) of 1.92 wt% exhibited the best methane combustion activity (*T*_50%_ = 320 °C and *T*_90%_ = 366 °C), which was better than that (*T*_50%_ = 337 °C and *T*_90%_ = 414 °C) over the Pd/KCC-1 sample, and significantly better than that over the 10.23CeO_2_/KCC-1 sample (results shown in Table 2 and Figure 5A).

The Pd dispersion on the supported Pd samples was determined using the CO-pulse method via the adsorption of CO. The apparent activation energies (*E*_a_), specific reaction rates, and turnover frequencies (TOF_Pd_) of the samples for methane combustion were calculated based on the activity data, Pd loadings, and Pd dispersion, as summarized in Table 2. The *E*_a_ (86 kJ/mol) obtained over 1.92Pd/9.68CeO_2_/KCC-1 was lower than that (*E*_a_ = 105 kJ/mol) over 1.96Pd/KCC-1 (see also Figure 5B). The specific reaction rate at 280 °C and the TOF_Pd_ at 280 °C decreased in the order of 1.92Pd/9.68CeO_2_/KCC-1 (0.39 mmol/(g_cat_ s) and 0.683 s^−1^) > 1.96Pd/KCC-1 (0.27 mmol/(g_cat_ s) and 0.390 s^−1^), providing further evidence for the enhanced methane oxidation performance by ceria modification on Pd/KCC-1. To investigate the effects of Pd doping and CeO_2_ modification on the intrinsic activity of the sample, the specific rate at 280 °C of our best sample is compared with that of other Pd-based catalysts reported in the literature (Table S1). Obviously, 1.92Pd/9.68CeO_2_/KCC-1 sample exhibited better specific reaction rate at 280 °C (0.39 μmol/(g_cat_ s)) than the 1.96Pd/KCC-1 sample, which was also higher than those (0.14–0.37 μmol/(g_cat_ s)) over 0.55Pd/ZrO_2_, Pd/NiCo_2_O_4_, Pd/CeO_2_@SiO_2_, 0.97Pd/3DOM Ce_0.7_Zr_0.3_O_2_, and Pd-NF/Al_2_O_3_, but lower than that (0.96 μmol/(g_cat_ s)) over Pd/NiCo_2_O_4_/NiAl_2_O_4_/*γ*-Al_2_O_3_ and that (0.75 μmol/(g_cat_ s)) over 0.44PtPd_2.2_/ZrO_2_.

Shown in Figure 5C are the long-term stability test results obtained over the 1.92Pd/9.68CeO_2_/KCC-1 sample for CH_4_ oxidation at 370 °C and SV = 20,000 mL/(g h). Apparently, there were no significant declines in the measured methane conversions after 40 h of on-stream reaction. In other words, the 1.92Pd/9.68CeO_2_/KCC-1 sample exhibited good catalytic stability under the adopted reaction conditions. In the actual methane-containing emissions, there is the co-presence of methane and water, in which water can exert a negative effect on activity of a catalyst [51]. Consequently, it is necessary to examine the effect of moisture with a certain concentration on CH_4_ combustion of the typical sample, and the results are shown in Figure 5D. CH_4_ conversions remained almost unchanged under the water-free condition. However, introducing 5.0 vol% water vapor into the reaction system gave rise to a decrease in activity (CH_4_ conversion decreased from 95 to 88%). After 5.0 vol% water vapor was removed from the feedstock, CH_4_ conversions were recovered to approximately 95% and maintained stably in the subsequent reaction time. The slight deactivation of the 1.92Pd/9.68CeO_2_/KCC-1 sample induced by water vapor addition was reversible. That is to say, 1.92Pd/9.68CeO_2_/KCC-1 possessed good water resistance. Additionally, we performed the H_2_O-TPD experiments of the samples, and their profiles are illustrated in Figure S8. All the samples exhibited water desorption peaks within the range of 100–300 °C. In previous studies, such water-induced inhibition has been linked to the reaction of PdO + H_2_O → Pd(OH)_2_, thereby reducing the active phase of PdO [52]. The selected KCC-1 support played an important role in alleviating this negative effect, enabling the inner-layer PdO to maintain high reactivity.

### 3.4. In Situ DRIFTS Analysis

**Figure 6:**
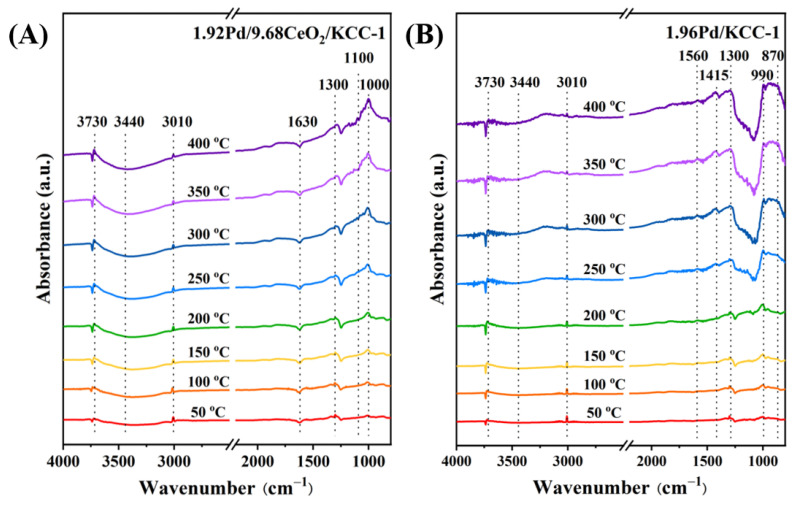
In situ DRIFTS spectra of methane combustion over the (**A**) 1.92Pd/9.68CeO_2_/KCC-1 and (**B**) 1.96Pd/KCC-1 samples under the conditions of (10,000 ppm CH_4_ + 20.0 vol% O_2_ + N_2_ (balance)), different temperatures (50–400 °C), and reaction time = 10 min.

The adsorption and desorption of methane also play a key role in determining methane oxidation. To further investigate the dissociation process of methane on the sample surface, in situ DRIFTS technique was employed. After 1 h of oxygen pretreatment at 300 °C, the in situ DRIFTS spectra of CH_4_ oxidation over 1.96Pd/KCC-1 and 1.92Pd/9.68CeO_2_/KCC-1 with increasing temperature are shown in Figure 6. The absorption band at 3010 cm^−1^ was attributed to asymmetric stretching vibration of the C–H bond in CH_4_; the one at 3730 cm^−1^ corresponded to vibration of the hydroxyl group [53]; the ones at 3440 and 1630 cm^−1^ originated from vibrations of the adsorbed H_2_O formed during the methane oxidation process; the one between 1100 and 1300 cm^−1^ was ascribed to vibration of the –OOH species; the one at 1000 cm^−1^ was due to vibration of the O_2_^−^ species; and the one at 1560 cm^−1^ was owing to vibration of the CO_3_^2−^ species [54]. Upon oxygen introduction, methane underwent a series of chemical reactions over the sample to be converted to –CH_3_O. During continuous oxygen supply, the intermediate products underwent deep oxidation to produce formate and carbonate [55]. These intermediates underwent complete oxidation, and the combustion process finally yielded to carbon dioxide and water. Concurrently, as the temperature rose, a gradual weakening in intensity of the methane absorption band was observed. By comparing the two catalysts, it can be observed that the catalyst doped with cerium oxide shows a faster change in the peak representing CO_3_^2−^, indicating that the intermediate products attached to the surface of the catalyst can decompose more quickly (i.e., the intermediate products are consumed at an increased rate), thereby enabling methane to completely convert into carbon dioxide and water. This result indicates that methane is first activated, resulting in the cleavage of its C–H bonds. Subsequently, formate and carbonate species were accumulated on the sample surface and further oxidized to CO_2_ and H_2_O [56]. The above characterization results confirm the good ability of 1.92Pd/9.68CeO_2_/KCC-1 to activate C-H bonds in methane. Figure S9 shows in situ DRIFTS spectra of methane combustion over the 1.92Pd/9.68CeO_2_/KCC-1 sample under the conditions of (10,000 ppm CH_4_ + 20.0 vol% O_2_ + N_2_ (balance)), different time (20–180 min), and 400 °C. It can be observed that the intensity of the individual absorption bands undergo no significant changes, further demonstrating the good catalytic stability of 1.92Pd/9.68CeO_2_/KCC-1.

**Figure 7:**
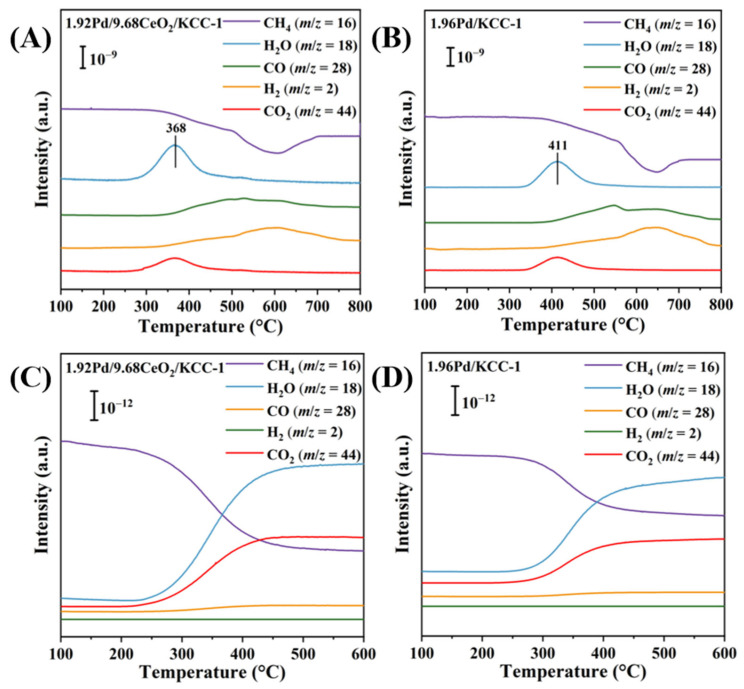
(**A**,**B**) CH_4_-TPR and (**C**,**D**) CH_4_-TPSR profiles of (**A**,**C**) 1.92Pd/9.68CeO_2_/KCC-1 and (**B**,**D**) 1.96Pd/KCC-1.

To investigate the effect of CeO_2_ doping on methane combustion activity, we conducted the CH_4_-TPR experiments of the 1.96Pd/KCC-1 and 1.92Pd/9.68CeO_2_/KCC-1 samples. As shown in Figure 7A,B, the intensity of the methane signal at 368 °C began to weaken, accompanied by CO_2_ generation, confirming that methane oxidation commenced around this temperature. Simultaneously collected signals reveal that the oxidation of methane on the sample surface yields CO_2_ and H_2_O as the final products. Following modification of the KCC-1-supported Pd sample with CeO_2_, the desorption peak due to water was shifted to a lower temperature (from 411 °C to 368 °C), which was associated with the reaction of the lattice oxygen in PdO of the 1.92Pd/9.68CeO_2_/KCC-1 sample that was stabilized by the adsorbed oxygen species formed at oxygen vacancies of CeO_2_, thereby generating more CO_2_. However, a slight increase in intensity of the signals due to CO and H_2_ after 400 °C was also observed, suggesting that methane dry reforming (CH_4_ + CO_2_ = CO + H_2_) [57] might occur on the sample surface.

It has been well-known that methane contains four identical and symmetrical C–H bonds with a high bond energy of 439 kJ/mol. Consequently, the activation of methane molecules requires high temperatures. We performed the CH_4_-TPSR experiments with the 1.92Pd/9.68CeO_2_/KCC-1 and 1.96Pd/KCC-1 samples, and their results are shown in Figure 7C,D. Obviously, methane consumption increased with the rise in temperature. Furthermore, methane consumption rate over 1.92Pd/9.68CeO_2_/KCC-1 was higher than that over 1.96Pd/KCC-1, and simultaneously, the generated CO_2_ and H_2_O amounts over 1.92Pd/9.68CeO_2_/KCC-1 were higher than those over 1.96Pd/KCC-1. The results indicate that methane undergoes essentially complete combustion over the samples (i.e., no byproducts such as CO or H_2_ are formed) [58,59]. Moreover, the onset temperatures for CO_2_ formation over the samples aligned with their catalytic activities in the oxidation of methane. This correlation was consistent with the CH_4_-TPR characterization results.

## 4. Conclusions

1.92Pd/9.68CeO_2_/KCC-1 and 1.96Pd/KCC-1 were prepared. Pd NPs were highly dispersed on the surface of 1.92Pd/9.68CeO_2_/KCC-1, and the synergistic interaction between CeO_2_ and PdO further enhanced catalytic activity. 1.92Pd/9.68CeO_2_/KCC-1 showed the outstanding catalytic performance for methane combustion, which was much better than that over 1.96Pd/KCC-1. In addition, the 1.92Pd/9.68CeO_2_/KCC-1 sample exhibited good high-temperature stability. The enhanced methane combustion performance of 1.92Pd/9.68CeO_2_/KCC-1 was mainly associated with the good dispersion of Pd species, the stabilization of the active Pd^2+^ species, and the generation of more reactive oxygen species.

## Figures and Tables

**Table 1:** Actual Pd and Ce contents, surface areas (*S*_BET_), average pore diameters, surface element compositions, and H_2_ consumption of the samples.

Sample	Actual Pd Content ^a^ (wt%)	Actual Ce Content ^a^ (wt%)	*S*_BET_ ^b^ (m^2^/g)	Average Pore Size ^b^ (nm)	Surface Element Composition ^c^			H_2_ Consumption ^d^ (μmol/g)
					Ce^3+^/(Ce^3+^ + Ce^4+^) Molar Ratio	O_ads_/O_latt_ Molar Ratio	Pd^2+^/(Pd^0^ + Pd^2+^) Molar Ratio	
KCC-1	–	–	555	12.81	–	–	–	–
1.96Pd/KCC-1	1.96	–	480	11.59	–	–	10.50	0.032
10.23CeO_2_/KCC-1	–	10.23	367	10.03	11.42	17.66	–	–
1.92Pd/9.68CeO_2_/KCC-1	1.92	9.68	304	9.43	24.16	20.13	14.22	0.171
1.44Pd/9.68CeO_2_/KCC-1	1.44	9.68	303	9.68	23.15	18.92	13.46	–
0.97Pd/9.68CeO_2_/KCC-1	0.97	9.68	301	9.95	22.90	18.01	12.49	–

^a^ Data were determined by the ICP–AES technique; ^b^ data were calculated by the BET method; ^c^ data were obtained by quantitatively analyzing the peaks in XPS spectra of the samples; ^d^ data were obtained by quantitatively analyzing the peaks in H_2_-TPR profiles of the samples.

**Table 2:** Catalytic activities, specific reaction rates at 280 °C, apparent activation energies (*E*_a_), Pd dispersion, and TOF_Pd_ at 280 °C of the samples for methane combustion.

Sample	*T*_10%_ (°C)	*T*_50%_ (°C)	*T*_90%_ (°C)	Specific Reaction Rate at 280 °C (μmol/(g_cat_ s))	*E*_a_ (kJ/mol)	Pd Dispersion ^a^ (%)	TOF_Pd_ (s^−1^)
1.96Pd/KCC-1	276	337	414	0.27	105	37.6	0.390
10.23CeO_2_/KCC-1	403	>500	>500	–	–	–	–
1.92Pd/9.68CeO_2_/KCC-1	265	320	366	0.39	86	31.7	0.683

^a^ Data were measured by the CO-pulsing method.

## Data Availability

Data will be made available on request.

## Supplementary Materials

The following supporting information can be downloaded at https://www.mdpi.com/article/10.3390/nano16040231/s1, S1. Content; S2. Chemicals; S3. Catalyst characterization procedures; Table S1: Catalytic activities (*T*_50%_ and *T*_90%_) and specific reaction rates at 280 °C for methane combustion over the 1.92Pd/9.68CeO_2_/KCC-1 sample presented in this work and the Pd-based catalysts reported in the literature; Figure S1: Additional SEM images of the (A) KCC-1 and (B) 1.96Pd/KCC-1 samples; Figure S2: Additional TEM images of the 1.92Pd/9.68CeO_2_/KCC-1 sample; Figure S3: Pd particle-size distributions of the 1.92Pd/9.68CeO_2_/KCC-1 and 1.96Pd/KCC-1 samples; Figure S4: Pd 3d XPS spectra of the *x*Pd/9.68CeO_2_/KCC-1 (*x* = 0.97, 1.44, and 1.92 wt%) samples; Figure S5: In situ CO-DRIFTS spectra of the 1.96Pd/KCC-1 sample; Figure S6: Methane conversion as a function of temperature for methane combustion over the *x*Pd/9.68CeO_2_/KCC-1 (*x* = 0.97, 1.44, and 1.92 wt%) samples at SV = 20,000 mL/(g h); Figure S7: Methane conversion as a function of temperature over the 1.92Pd/*y*CeO_2_/KCC-1 (*y* = 5.27, 9.68, and 18.81 wt%) samples for methane combustion at SV = 20,000 mL/(g h); Figure S8: H_2_O-TPD profiles of the 1.96Pd@KCC-1 and 1.92Pd/9.68CeO_2_/KCC-1 samples; Figure S9: In situ DRIFTS spectra of methane combustion over the 1.92Pd/9.68CeO_2_/KCC-1 sample under the conditions of (10,000 ppm CH_4_ + 20.0 vol% O_2_ + N_2_ (balance)), different time (20–180 min), and 400 °C.
