# Supplementary material for: Boosting Methane Combustion Performance and Stability of Spherical Mesoporous Silica (KCC-1)-Supported Pd Catalysts by Modifying with CeO2
Source: Nanomaterials (Basel). 2026 Feb 11;16(4):231. doi: 10.3390/nano16040231 (PMC12943732; doi:10.3390/nano16040231)
Supplement: Supplementary file 1 [file nanomaterials-16-00231-s001.zip › nanomaterials-4122259-supplementary.pdf]

Supplementary Material

Boosting Methane Combustion Performance and Stability of Spherical Mesoporous Silica (KCC-1)-Supported Pd Catalysts by Modifying with CeO_2

Kaien Feng [1], Jinxiong Tao [1], Zhiquan Hou [1,2], Yuxi Liu [1,2], Jiguang Deng [1,2], Lu Wei [1,2], Zhen Wei [1,2], Lin Jing [1,2], Hongxing Dai [1,2,*]

[1] Beijing Key Laboratory for Green Catalysis and Separation, State Key Laboratory of Materials Low-Carbon Recycling, Laboratory of Catalysis Chemistry and Nanoscience, Department of Chemical Engineering and Technology, College of Materials Science and Engineering, Beijing University of Technology, Beijing 100124, China

[2] Key Laboratory of Beijing on Regional Air Pollution Control, College of Environmental Science and Engineering, Beijing University of Technology, Beijing 100124, China

* Correspondence: hxdai@bjut.edu.cn

S1. Content

Item	Page
**Chemicals**	3
**Catalyst characterization procedures**	3–5
**Table S1**	6
**Fig. S1**	7
**Fig. S2**	7
**Fig. S3**	8
**Fig. S4**	9
**Fig. S5**	9
**Fig. S6**	10
**Fig. S7**	10
**Fig. S8**	11
**Fig. S9**	11

S2. Chemicals:

All the reagents were commercially obtained without purification. Tetraethyl orthosilicate (TEOS), hexadecyltrimethylammonium bromide (CTAB), 1-pentanol (Beijing Honghu Union Chemical Reagent, 99.5 %), methanol (CH_3OH, Tianjin Fuchen Chemical Reagent, 99.5 %), urea (CH_4N_2O, Beijing Honghu Union Chemical Reagent, 99 %), cerium nitrate hexahydrate ($Ce(NO_3)_3·6H_2O$, Aladdin, 99.95 %), cyclohexane (C_6H_{12}, Tianjin Fuchen Chemical Reagent, 99.5 %), ammonia solution ($NH_3·H_2O$, Tianjin DaMao Chemical Reagent Factory, 28 %), and platinum chloride (Aladdin Company). All the gases used in this work were purchased from the Beijing Beiyang and Yangyuan Commerce and Trade Center Company.

S3. Catalyst characterization procedures:

The actual metal contents in the as-obtained catalysts were determined using the inductively coupled plasma–atomic emission spectroscopic (ICP–AES) technique on a Thermo Electron IRIS Intrepid ER/S spectrometer. Each sample was dissolved in a mixture of concentrated HCl (37 wt%) and HNO_3 (75 wt%) aqueous solutions with a volumetric ratio of 3 : 1 prior to analysis.

Crystal structures of the as-obtained catalysts were characterized by means of the X-ray diffraction (XRD) technique on a Bruker/AXS D8 Advance diffractometer, with Cu Kα radiation and nickel filter (λ = 0.15406 nm). The

scanning conditions were set with a step size of 0.02 ° and a time per step of 1 s.

The scanning electron microscopic (SEM) and transmission electron microscopic (TEM) images of the as-obtained samples were recorded on a Gemini Zeiss Supra 55 apparatus (operated at 10 kV) and a JEOL JEM-2010 instrument. High-angle annular dark field–scanning transmission electron microscopy (HAADF–STEM) and EDX element mappings of the typical sample were also taken on the Cs-corrected cold field-emission FEI G2 80-200/Chemi-STEM operated at an accelerating voltage of 200 kV.

Laser Raman spectra of the samples were determined on a Bruker RFS/100 Raman spectrometer. A red laser (532 nm) was used as excitation source.

The measurements of specific surface areas, pore volumes, and pore diameters were carried out on a Micromeritics ASAP 2020 analyzer via N_2 adsorption at –196 °C. All the samples were degassed at 200 °C for 6 h under vacuum prior to measurement. Surface areas of the samples were determined using the Brunauer–Emmett–Teller (BET) method.

The X-ray photoelectron spectroscopy (XPS, Thermo Fisher Scientific ESCALAB 250 Xi spectrometer) was used to determine the Ce 3d, Pd 3d, O 1s, Si 2p, and C 1s binding energies (BEs) of the surface species with Mg Kα ($h\nu$ = 1253.6 eV) as the excitation source. All the samples were pretreated in air at 300 °C for 1 h. The C 1s signal at BE = 284.6 eV was taken as reference for BE calibration.

Fourier transform infrared spectroscopic (FT-IR) spectra of the samples were recorded on a Tensor II (Bruker) FT-IR spectrometer with a liquid-nitrogen-cooled MCT detector. In the case of CO adsorption and desorption, before the experiments, 30 mg of the as-obtained sample was loaded into a high-temperature IR cell with a KBr window. Before CO adsorption, the sample was reduced in situ at 200 °C in 10 vol% H_2/N_2 for 0.5 h and then purged with N_2 at 300 °C for 0.5 h. Subsequently, the sample was cooled to 30 °C, and the background spectrum was recorded. After that, a 10 vol% CO/N_2 flow (30 mL/min) was passed through the IR cell for 0.5 h. A N_2 flow (30 mL/min) was passed through the IR cell, and the FT-IR spectra of the samples were recorded in the N_2 flow at 30 °C and different time, with an accumulation of 32 scans and a spectrum resolution of 4 cm^{-1}.

Hydrogen temperature-programmed reduction (H_2-TPR) experiments of the samples were carried out on a chemical adsorption analyzer (Autochem II 2920, Micromeritics). Before TPR measurement, ca. 0.05 g of the sample (40–60 mesh) was loaded to a fixed-bed U-shaped quartz microreactor (i.d. = 4 mm) and pretreated in a 20 vol% O_2/He flow of 30 mL/min at 300 °C for 1 h. After being cooled to room temperature (RT) at the same atmosphere, the sample was purged in a He flow of 30 mL/min for 30 min. The pretreated sample was exposed to a flow (30 mL/min) of 10 vol% H_2/Ar mixture and heated at a ramp of 10 °C/min from RT to 900 °C. The alteration in H_2 concentration of the effluent was monitored online by the chemical adsorption analyzer. The reduction peak was calibrated against that of the complete reduction of a known standard powdered CuO (Aldrich, 99.995 wt%).

Methane programmed temperature reduction (CH_4-TPR) experiments of the samples were performed on a chemisorption analyzer (Autochem II 2920, Micromeritics) and a mass spectrometer (OmniStar GSD320, Pfeiffer Vacuum). Prior to each test, 50 mg of catalyst was treated in an O_2 flow of 30 mL/min at 300 °C for 1 h. After cooling to RT, a mixed gas stream of (10,000 ppm CH_4 + He (balance)) was allowed to pass through the catalyst for an additional 30 min, and then the temperature was raised to 900 °C at a ramp of 10

°C/min. On-line mass spectrometer was used to record the signals of methane (*m*/*z* =16 or 15), H_2O (*m*/*z* = 18), CO (*m*/*z* = 28), CO_2 (*m*/*z* = 44), and H_2 (*m*/*z* = 2).

Metal dispersion was measured using chemisorption (AutoChem II 2920, Micromeritics). The sample was reduced in a H_2 flow (30 mL/min) at 200 °C for 2 h, purged with a He flow (30 mL/min) for 1 h, and cooled to 50 °C. Then, it was saturated with pulses of CO. The uptake of CO during the chemisorption was measured by a TCD.

H_2O temperature-programmed desorption (H_2O-TPD) measurements were performed. Prior to the analysis, the sample was saturated by water in the flow (20 mL/min) of 4.5 vol% H_2O/N_2 at 100 °C for 1 h, and then purged with a He flow of 20 mL/min at 100 °C for 1 h to remove the physically adsorbed and condensed water on the surface of sample. Finally, the TPD measurements were carried out by increasing the temperature at a rate of 10 °C/min from 100 to 600 °C and the signal of H_2O (*m*/*z* = 18) was recorded by the mass spectrometer (OmniStar GSD320, Pfeiffer Vacuum).

Methane temperature-programmed surface reaction (CH_4-TPSR) experiments of the samples were performed on a chemisorption instrument (Autochem II 2920, Micromeritics) and a mass spectrometer (OmniStar GSD320, Pfeiffer Vacuum). Prior to each test, 50 mg of the catalyst was first treated in a 30 mL/min O_2 flow at 300 °C for 1 h to remove the impurity gases from the surface. After cooling to RT, a (10,000 ppm CH_4 + 20.0 vol% O_2 + N_2 (balance)) mixture was allowed to pass through the catalyst. After the baseline was stabilized, the temperature was then increased at a ramp rate of 10 °C /min from RT to 900 °C. On-line mass spectrometer was used to record the signals of methane (*m*/*z* = 16 or 15), H_2O (*m*/*z* = 18), CO (*m*/*z* = 28), CO_2 (*m*/*z* = 44), O_2 (*m*/*z* = 32), and H_2 (*m*/*z* = 2).

In situ diffuse reflectance infrared Fourier transform spectroscopic (in situ DRIFTS) spectra of the samples were recorded on a Tensor II (Bruker)) FT-IR spectrometer with a liquid-nitrogen-cooled MCT detector. In the in situ DRIFTS studies, 30 mg of the sample was loaded to a high-temperature IR cell with a ZnSe window, a N_2 flow (30 mL/min) was passed through the IR cell at 30 °C for 20 min, and the background spectrum was recorded. Subsequently, the N_2 flow was switched to the (10,000 ppm CH_4 + 20.0 vol% O_2 + N_2 (balance)) mixture flow of 16.67 mL/min at elevated temperatures from 50 to 100, 150, 200, 250, 300, 350 or 400 °C, and the in situ DRIFTS spectra were recorded when the sample was kept at the given temperature for 30 min. DRIFTS spectra were recorded with an accumulation of 32 scans and a spectrum resolution of 4 cm^{-1}.

Table S1. Catalytic activities ($T_{50\%}$ and $T_{90\%}$) and specific reaction rates at 280 °C for methane combustion over the 1.92Pd/9.68CeO$_2$/KCC-1 sample presented in this work and the Pd-based catalysts reported in the literature.

Sample	Reactant mixture	SV (mL/(g h))	$T_{50\%}$ (°C)	$T_{90\%}$ (°C)	Specific reaction rate at 280 °C (μmol/(g_{cat} s))	Ref.
1.92Pd/9.68CeO_2/KCC-1	1 vol% CH_4 + 20 vol% O_2 + N_2 (balance)	20,000	320	366	0.39	This work
0.55Pd/ZrO_2	2.5 vol% CH_4 + 20 vol% O_2 + N_2 (balance)	20,000	369	440	0.32	[14]
0.44Pt$Pd_{2.2}$/ZrO_2	2.5 vol% CH_4 + 20 vol% O_2 + N_2 (balance)	20,000	345	408	0.75	[14]
Pd/$NiCo_2O_4$	1 vol% CH_4 + 20 vol% O_2 + N_2 (balance)	30,000	337	400	0.14	[17]
Pd/$NiCo_2O_4$/$NiAl_2O_4$/γ-Al_2O_3	1 vol% CH_4 + 20 vol% O_2 + N_2 (balance)	30,000	300	345	0.96	[17]
Pd/CeO_2@SiO_2	1 vol% CH_4 + 20 vol% O_2 + N_2 (balance)	30,000	360	405	0.19	[39]
0.97Pd/3DOM $Ce_{0.7}Zr_{0.3}O_2$	1 vol% CH_4 + 20 vol% O_2 + N_2 (balance)	20,000	330	380	0.30	[49]
Pd-NF/Al_2O_3	1 vol% CH_4 + 20 vol% O_2 + N_2 (balance)	20,000	339	390	0.37	[58]

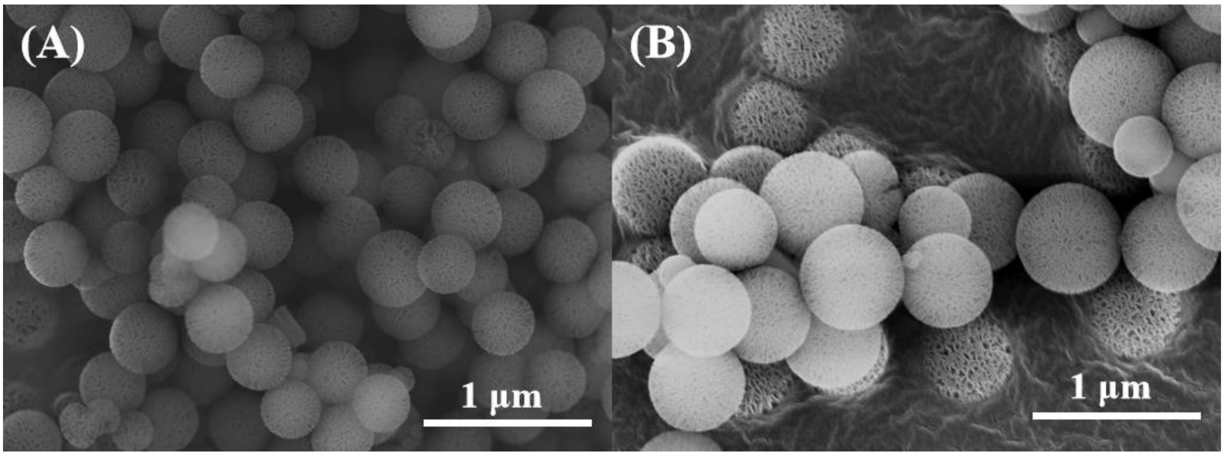

Figure S1. Additional SEM images of the (A) KCC-1 and (B) 1.96Pd/KCC-1 samples.

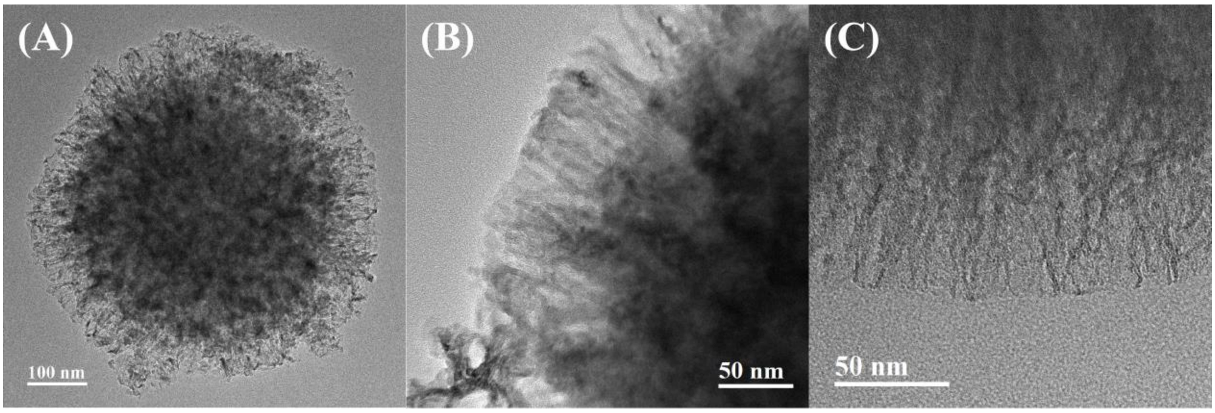

Figure S2. Additional TEM images of the 1.92Pd/9.68CeO2/KCC-1 sample.

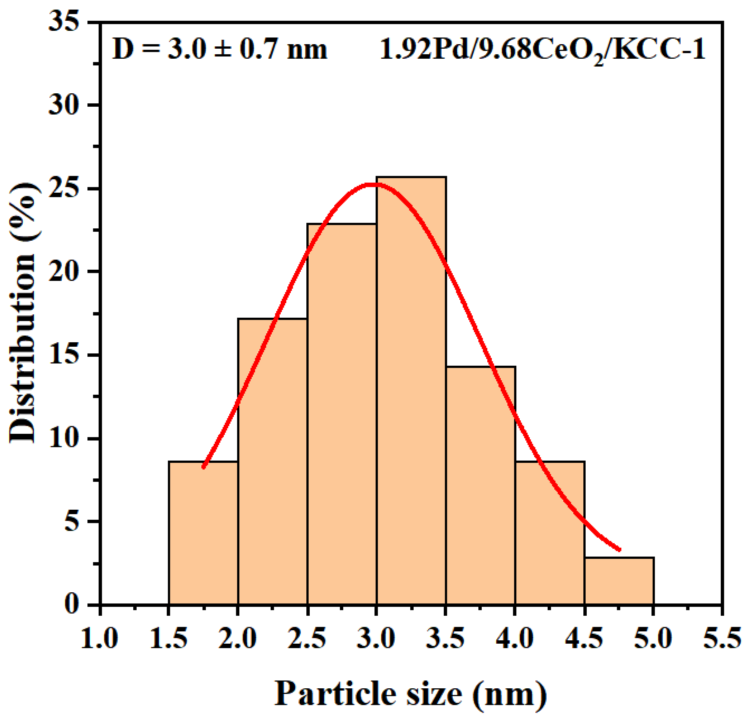

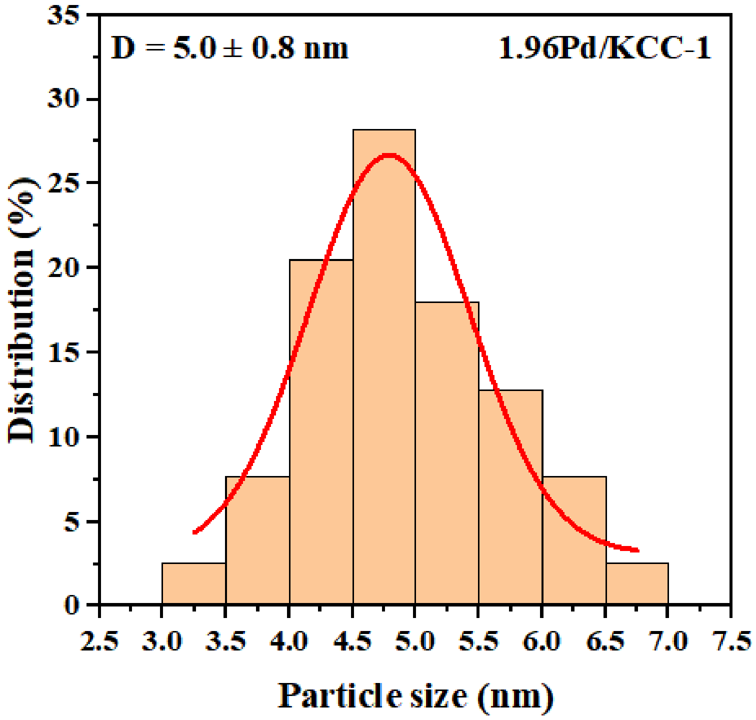

Figure S3. Pd particle-size distributions of the 1.92Pd/9.68CeO_2/KCC-1 and 1.96Pd/KCC-1 samples.

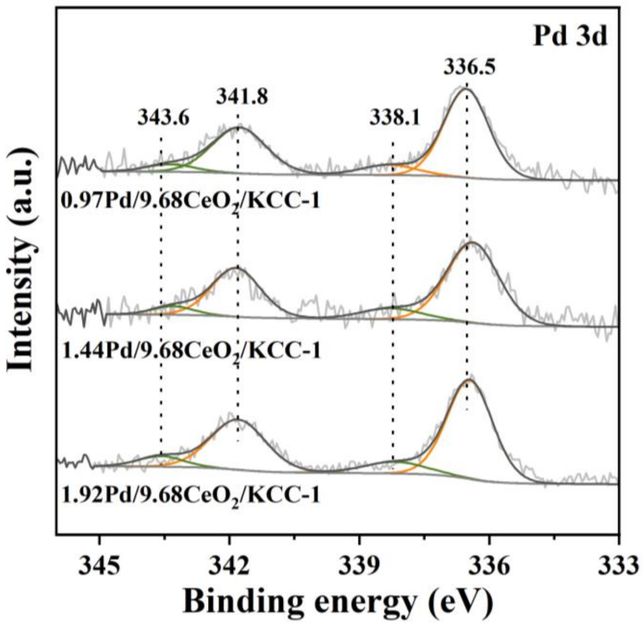

Figure S4. Pd 3d XPS spectra of the *x*Pd/9.68CeO_2/KCC-1 (*x* = 0.97, 1.44, and 1.92 wt%) samples.

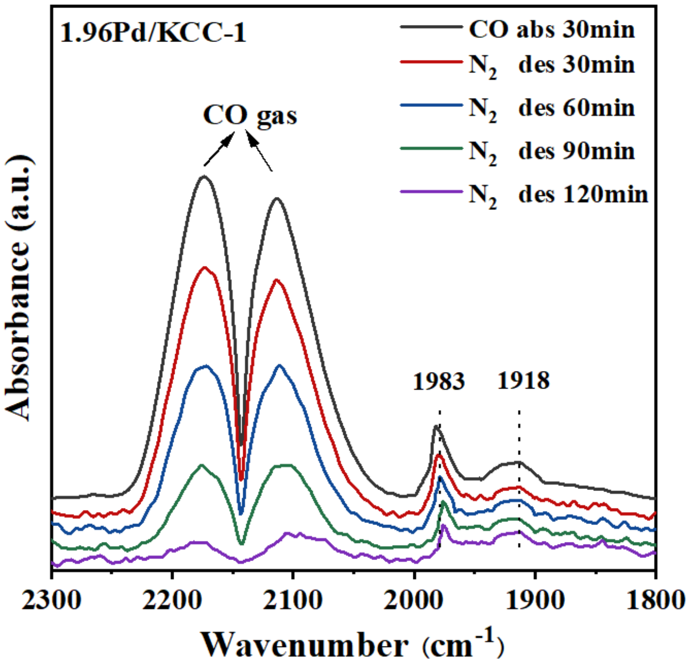

Figure S5. In situ CO-DRIFTS spectra of the 1.96Pd/KCC-1 sample.

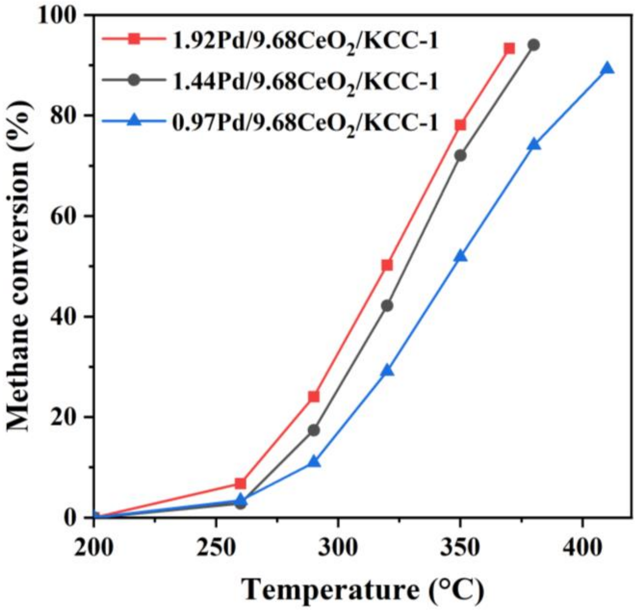

Figure S6. Methane conversion as a function of temperature over the xPd/9.68CeO_2/KCC-1 (x = 0.97, 1.44, and 1.92 wt%) samples for methane combustion at SV = 20,000 mL/(g h).

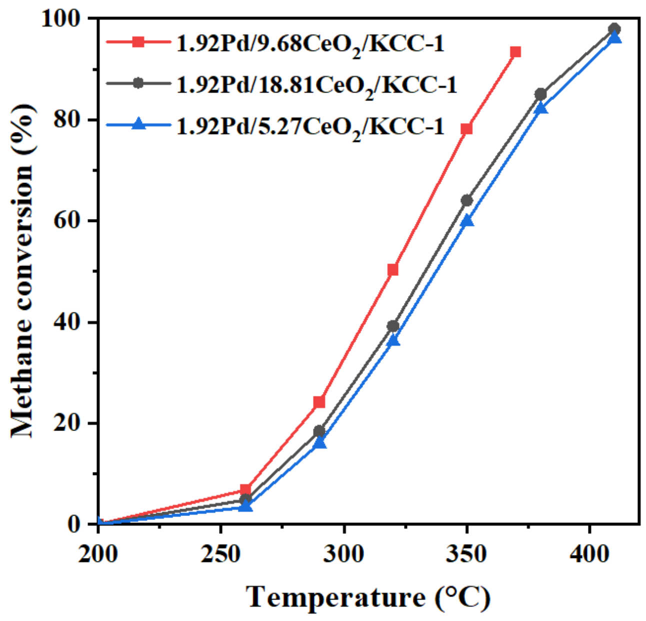

Figure S7. Methane conversion as a function of temperature over the 1.92Pd/yCeO2/KCC-1 (y = 5.27, 9.68, and 18.81 wt%) samples for methane combustion at SV = 20,000 mL/(g h).

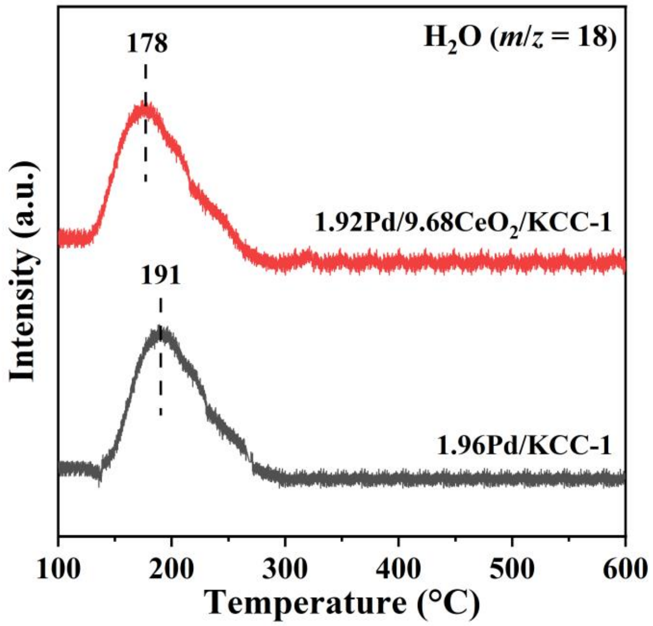

Figure S8. H_2O-TPD profiles of the 1.96Pd@KCC-1 and 1.92Pd/9.68CeO2/KCC-1 samples.

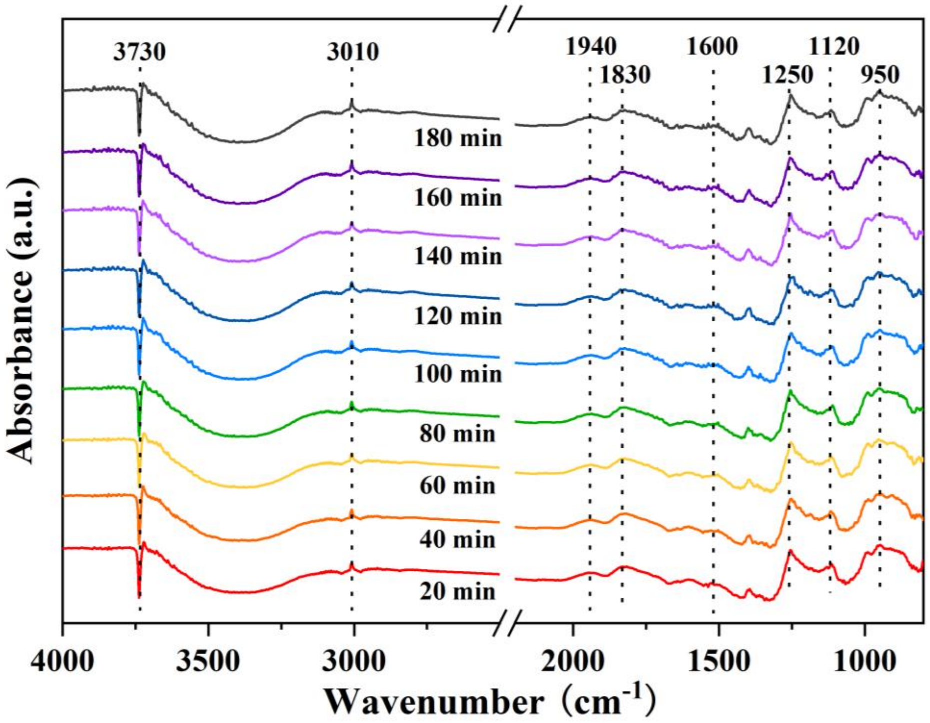

Figure S9. In situ DRIFTS spectra of methane combustion over the 1.92Pd/9.68CeO_2/KCC-1 sample under the conditions of (10,000 ppm CH_4 + 20.0 vol% O_2 + N_2 (balance)), different time (20–180 min), and 400 ^{o}C.
